# Signaling pathway intervention in premature ovarian failure

**DOI:** 10.3389/fmed.2022.999440

**Published:** 2022-11-25

**Authors:** Xuefeng Bai, Shaowei Wang

**Affiliations:** Department of Gynecology and Obstetrics, Beijing Hospital, National Center of Gerontology, Institute of Geriatric Medicine, Chinese Academy of Medical Sciences, Beijing, China

**Keywords:** premature ovarian failure, PI3K, mTOR, PTEN, signaling pathway

## Abstract

Premature ovarian failure (POF) is a multifactorial disease that refers to the occurrence of secondary amenorrhea, estrogen decrease, and gonadotropin increase in women under the age of 40. The prevalence of POF is increasing year by year, and the existing instances can be categorized as primary or secondary cases. This disease has adverse effects on both the physiology and psychology of women. Hormone replacement therapy is the recommended treatment for POF, and a multidisciplinary strategy is required to enhance the quality of life of patients. According to recent studies, the primary mechanism of POF is the depletion of ovarian reserve function as a result of increased primordial follicular activation or primordial follicular insufficiency. Therefore, understanding the processes of primordial follicle activation and associated pathways and exploring effective interventions are important for the treatment of POF.

## Introduction

Premature ovarian failure (POF), also known as premature menopause, is a condition in which a woman experiences amenorrhea before the age of 40 due to the cessation of ovarian function. POF can lead to infertility in women with physiological or psychological problems (1, 2). Genetic, immunological, metabolic, viral, and medicinal variables all have a role in the etiology of POF (3). Studies have shown that the daughters of POF patients are six times more likely to suffer from the disease than normal peers, and the treatment of diseases such as malignancies of the reproductive system and endometriosis often causes secondary POF (4). Abnormalities in some signaling pathways can also result in POF. To improve patients' quality of life and maintain their fertility, we should learn the processes of primordial follicle activation and oocyte death and explore the corresponding therapeutic prevention or therapy techniques (5). The purpose of this review is to provide an overview of the signaling pathways that play significant roles in follicular activation.

The ovary is an essential part of the female reproductive system that plays a crucial role in the growth and fertility of the reproductive system by producing mature oocytes and secreting many kinds of hormones (6). The primordial follicle, which begins to develop shortly after birth (7), is the reproductive unit of the mammalian ovary (8).

The primordial follicular pool, which is the only source of germ cells in female mammals, is formed by a vast number of primordial follicles that are distributed across the ovary's periphery (9). Females have about 10^6^ primordial follicles at birth, but only a small percentage of them mature into oocytes; the remainder are either kept dormant or disappear. One of the factors that influence the length of a woman's reproductive life is the balance between the primordial follicle's dormant, active, and apoptotic stages (10). Ninety-nine percent of the primordial follicles in women die as they age, and menopause occurs when there are less than 10^3^ primordial follicles left in the body (11). A woman is thought to have POF if this condition occurs before the age of 40.

Long-term hormone replacement therapy (HRT), which uses estrogen, progesterone, melatonin and other hormone to alleviate menopausal symptoms caused by POF, is the current preferred treatment (12, 13). HRT can be administered through a variety of methods, and the dose schedule is becoming more personalized (3). However, several clinical studies have revealed that people who receive HRT have a higher long-term risk of developing breast cancer, heart disease, stroke, and other disorders (14). In patients with reproductive malignancies, surgery can result in iatrogenic POF. Some early-stage patients may receive HRT while preserving fertility to prevent iatrogenic POF (15, 16). To preserve fertility, these patients can use drugs such as GnRH to promote ovulation and cryopreserve them before surgery, which is well developed (17, 18). In recent years, stem cells and their secreted cytokines or exosomes have been discovered to enhance the milieu of ovarian tissue, control inflammation, and encourage the growth of follicles. However, successful treatment has only been accomplished in laboratory animals (19).

## Follicular activation and its pathways

Activation of primordial follicles determines oocyte growth and development, as well as the differentiation and proliferation of surrounding somatic and granulosa cells, both of which are essential for the ovulation process (11). To provide new perspectives on the treatment of POF, we need to comprehend the crucial factors affecting human follicular activation and the mechanisms that control follicular activation. We also need to explore the mechanisms that control awakening and the early development of dormant follicles to understand the process of follicular development and apoptosis and propose targeted interventions to promote oocyte maturation. This is a crucial step in identifying, treating, and preventing POF (20).

**Figure 1:**
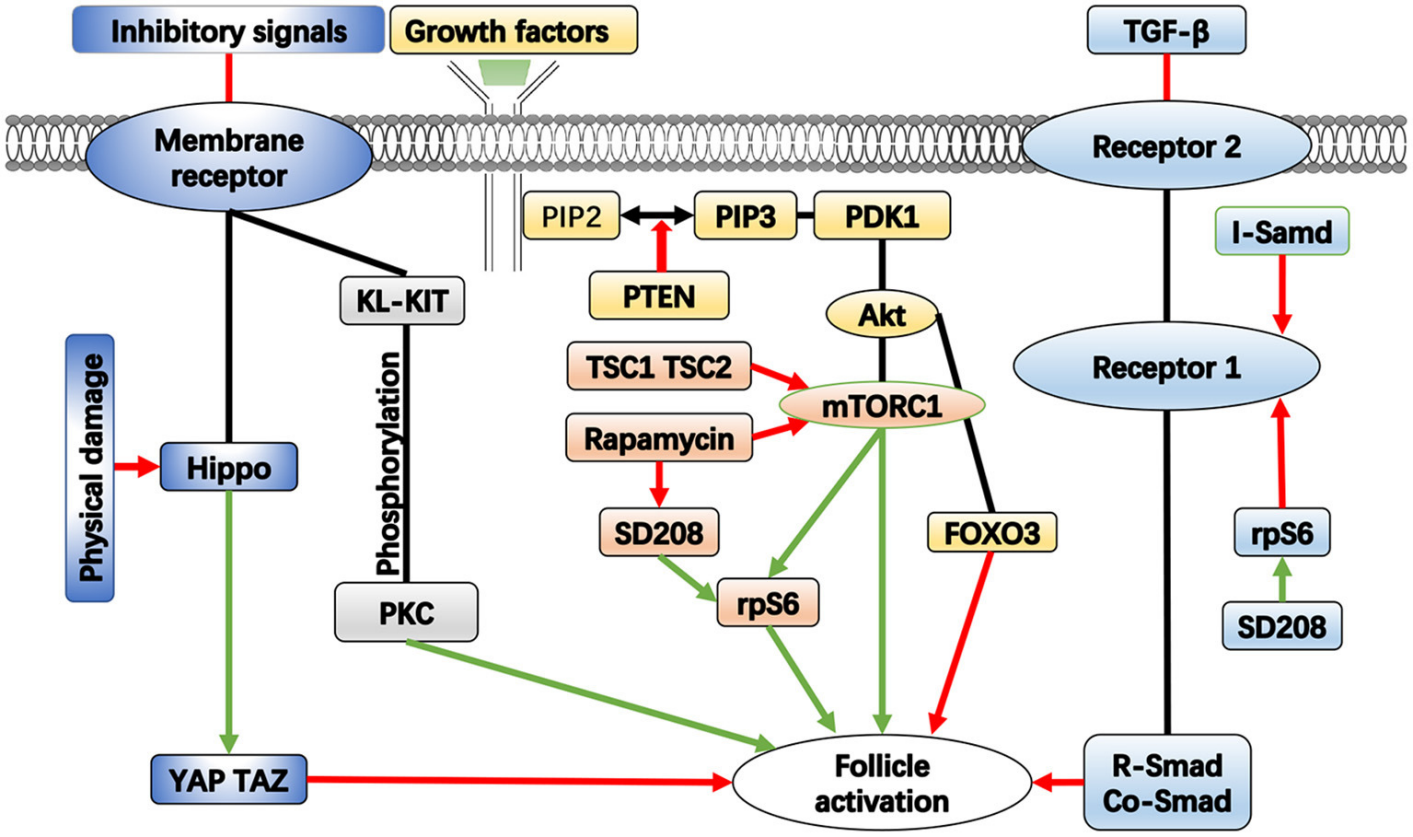
The important pathways in the activation of follicle. Green arrows represent enhancement, red arrows represent inhibition.

The primordial follicles are maintained in a dormant state by a certain inhibitory system, which is achieved by a combination of signaling pathways and molecular processes. Studies have shown that this system is necessary for the preservation of the dormant follicle pool (10). The current research on this system mainly focuses on follicle activation signaling pathways and related molecules, such as phosphatidylinositol 3 kinase (PI3K) and mammalian target of rapamycin (mTOR) (11). Loss of molecular function in any of these pathways has the potential to cause premature and irreversible activation of the primordial follicle pool, leading to depletion of dormant follicle reserves and POF (Figure 1). Further, the influence of the ovarian environment needs to be delineated. Some scholars have studied the effect of extracellular matrix on the activation of primordial follicles (11).

### PI3K/PTEN/Akt/Foxo3 pathway

Although the mechanism of primordial follicle activation is not fully understood, evidence shows that the phosphatidylinositol 3-kinase/protein kinase B pathway (PI3K/Akt) plays a key role in this process. Cell motility, survival, differentiation, growth, and intracellular transport are all regulated by PI3K, which catalyzes the phosphorylation of phosphatidylinositol (21). Protein kinase B (PKB), also known as Akt, is a general term for three serine/threonine-specific protein kinases that play important roles in a variety of cellular processes, including cell apoptosis, proliferation, transcription, and migration (22). The PI3K/Akt pathway is composed of various signaling molecules, such as kinases, phosphatases, and transcription factors, that establish intracellular signaling cascades. It is a classic intracellular signaling pathway that not only regulates cell proliferation, survival, and migration but is also involved in the activation of primordial follicles in the ovary (23). Akt is a crucial kinase in primordial follicle activation; it has a variety of substrates that are expressed in both oocytes and granulosa cells of the human ovary, playing both direct and indirect roles in follicle activation (24). Among the various substrates of Akt, the Foxo3 protein was first found to control the activation pathway of primordial follicles, which is encoded by the Foxo3 gene. Experiments have shown that in mice deficient in the Foxo3 gene, all dormant follicles are prematurely activated in the pubertal ovary, and when the active Foxo3 gene is expressed in mouse oocytes, the development of oocytes and follicles is delayed; this means that normal expression of the Foxo3 gene can inhibit follicular development and maintain a dormant state of follicles (25). Goldbraikh et al. highlighted PI3K/Akt/Foxo3 signaling as the central pathway controlling growth and metabolism in all cells (26). The PI3K pathway can be inhibited by the deletion of the phosphatase and tensin homolog gene PTEN on human chromosome 10. The PTEN gene encodes a specific phospholipid phosphatase that selectively dephosphorylates phosphatidylinositol substrates and negatively regulates intracellular phosphatidylinositol triphosphate (PIP3) to generate diphosphate products (23). This inhibits cell growth, proliferation, and survival signaling pathways mediated by PI3K. This function is significant because it suppresses the Akt signaling pathway, which is crucial for controlling the growth, survival, and migration of cells. Due to the above functions, PTEN can participate in the regulation of the cell cycle, prevent the rapid growth and division of cells, induce apoptosis, inhibit the adhesion of cells to the surroundings, and prevent the formation of new blood vessels. PTEN can also maintain the stability of cell genetic information. All of these functions help prevent the occurrence of malignant cell proliferation. As a result, numerous anticancer drugs were designed to target the PTEN gene because it was previously considered to be of great significance for the treatment of malignant tumors (27).

In the process of follicle development, PTEN has an inhibitory effect on the PI3K/Akt pathway, maintaining primordial follicles in a dormant state for a long time. When the inhibition of PTEN is removed, PI3K is activated in primordial follicles, converting phosphatidylinositol diphosphate (PIP2) to PIP3, thereby stimulating phosphatidylinositol-dependent kinase 1 (PDK1), and activating Akt. This causes the downstream Foxo3 protein to be phosphorylated, resulting in its loss of transcriptional activity and relocation from the nucleus to the cytoplasm for destruction, which activates primordial follicles (28). As a negative regulator of PI3K, PTEN can lead to inhibition of the PI3K signaling pathway. Studies have shown that when the expression of the PTEN gene is inhibited due to the enhanced PI3K signaling pathway in mammals, dormant primordial follicles are activated. When the PTEN gene is knocked out from the oocytes of mouse primordial follicles, overgrowth of primordial follicles and premature activation of the entire primordial follicle pool can be observed at puberty, which also leads to follicle depletion in early adulthood and POF in mice (29). Han et al. compared the granulosa cells of POF patients with those of healthy individuals and found that interleukin-4 (IL-4) levels were higher in POF patients. IL-4 can inhibit the growth of granulosa cells by activating PI3K/Akt pathway (30). Liu et al. found that exosomes from stem cells can enhance the expression of PI3K/Akt pathway, increase the proliferation rate of granulosa cells, and improve ovarian function in a POF mouse model (31). Li et al. (32) found that quercetin attenuated cyclophosphamide-induced follicle loss by preventing the phosphorylation of PI3K/Akt/Foxo3 pathway members and maintaining the anti-Müllerian hormone level through reduced apoptosis in growing follicles (32). These findings imply that the PI3K/PTEN/Akt/Foxo3 cascade in oocytes is essential for the activation of primordial follicles (10).

### mTOR pathway

As a downstream molecule of the PI3K/Akt pathway, mammalian target of rapamycin (mTOR) signaling is a crucial intracellular signaling pathway that regulates the cell cycle and has received extensive attention (33). It is a member of the PI3K-related kinase family and is linked to other proteins. mTOR is the core component of two protein complexes known as mTOR protein complex 1 (mTORC1) and mTOR protein complex 2 (mTORC2), two protein complexes with different structures and functions. The two complexes have distinct downstream targets that regulate distinct cellular activity. As a highly conserved serine/threonine kinase among these two complexes, mTOR not only regulates cell growth, proliferation, survival, migration, and other activities but also participates in the regulation of protein synthesis, cell autophagy, gene transcription, and other processes (34). As a core element of mTORC2, mTOR also acts as a tyrosine kinase that influences cell metabolism and survival. It not only integrates information from upstream molecules, such as insulin, growth hormones, amino acids, and other substances, but also perceives the nutrition, oxygen, and energy levels of the cell. This route is a major metabolic and physiological regulator of mammals and is crucial for the health of the liver, muscles, brain, and other organs (35). Previous research has shown that the mTOR pathway's overactivation plays a significant role in the development of cancer because of its impact on proteins. In lung cancer, prostate cancer, breast cancer, and other malignant tumors, the activity of mTOR has been found to be significantly increased (36).

The most significant of the various reasons for elevated mTOR activity is the mutation of the PTEN gene. Normally, PTEN inhibits mTOR signaling by blocking the activity of PI3K, an upstream effector of mTOR. When the inhibitory impact of PTEN is weakened, mTOR promotes cell cycle progression, boosts proliferation, and prevents autophagy (37). Zhang et al. (38) found that histone deacetylase 6 (HDAC6) may play an indispensable role in balancing the maintenance and activation of primordial follicles through mTOR signaling in mice. HDAC6 is a microtubule-associated deacetylase that predominantly functions in the cytoplasm by deacetylating various substrates to regulate cell migration and motility. The expression level of mTOR in the follicle and the activity of PI3K in the oocyte of the follicle can be simultaneously upregulated by inhibiting HDAC6, which can also promote the activation of limited primordial follicles (38). Studies have shown that during the transition from primordial to primary follicles, glycolysis in granulosa cells is enhanced and can increase the expression of the mTOR signaling pathway, thereby promoting the activation of primordial follicles (39).

As an inhibitor of mTOR, rapamycin can bind to the FKBP12 protein, an intracellular receptor. By directly interfering with the FKBP12 rapamycin-binding domain of mTOR, the FKBP12–rapamycin combination inhibits the activity of mTOR (40). Among the two mTOR complexes, only mTORC1 is responsive to rapamycin. In mammals, mTORC1 promotes preprotein synthesis, ribosome formation, and cell growth by activating ribosomal protein S6 (RPS6) and eukaryotic translation initiation factors 4E (4E-BPs), which in turn positively regulates cell growth and proliferation (41). The activity of mTORC1 is negatively regulated by a heterodimeric complex composed of two protein molecules. The two protein molecules are tuberous sclerosis complex 1 (tsc1) and tuberous sclerosis complex 2 (tsc2), which are the products of the tumor suppressor genes tsc1 and tsc2. Tsc1 keeps tsc2 from deterioration and ubiquitination to maintain its stability. They combine to generate a heterodimeric complex that prevents mTORC1 from being activated (42). Similar to the absence of PTEN in mouse oocytes, when tsc1 and tsc2 are specifically deleted, premature activation of primordial follicles occurs in mouse ovaries, resulting in an overall activation of primordial follicles around puberty, causing follicular depletion in early adulthood (43). This finding implies that excessive activation of mTORC1 signaling accelerates the activation of primordial follicles, and rapamycin, an inhibitor of mTORC1, can inhibit the development of primordial follicles and maintain the size of the follicular pool by blocking this signaling pathway. After treating POF mice with electroacupuncture therapy, He et al. (44) found that this method can promote the expression of mTOR pathway, induce the proliferation of ovarian cells, and restore the estrous cycle in mice (44). Shi et al. (45) designed a new biomaterial that moderately inhibits the mTOR pathway and prevents premature follicle activation, thereby delaying ovarian aging (45).

### Hippo pathway

The Hippo signaling pathway is composed of a series of conserved kinases and is an important signaling pathway regulator. It can control the size of an organ by regulating cell proliferation and apoptosis, and has the ability to inhibit cell growth (46). In mammals, the membrane protein receptor is upstream of the Hippo signaling pathway and is a receptor for extracellular growth inhibition signals. Once the extracellular growth inhibition signal is activated, it initiates a series of kinase cascade phosphorylation reactions, eventually resulting in the phosphorylation of the downstream transcriptional co-activators YAP and TAZ. The cytoskeletal proteins will bind to the phosphorylated transcriptional co-activators, forcing them to remain in the cytoplasm and decrease the activity of the nucleus, accomplishing regulation of organ growth and volume (47).

Hippo signaling is also impacted by changes in cellular connections, shape, and polarity. Intercellular tension in organs is altered when organs are physically damaged, accompanied by inhibition of the Hippo signaling pathway accumulation of co-activators in the nucleus. This series of modifications affects organ size and controls cell proliferation. Cell proliferation, survival, and growth are promoted when Hippo signaling is inhibited (48).

The Hippo signaling system has been demonstrated in mouse models to play a function in controlling cell proliferation and death by preserving organ volume at the ideal size by inhibiting cell growth (49). Genes connected to the Hippo signaling system are expressed in follicles at different stages of mice and human ovaries. Fragmentation of the ovary temporarily stimulates follicle development and reduces the phosphorylation of YAP. The disruption of mouse ovaries promotes dynamic changes in actin and disturbs the Hippo signaling pathway, increasing the growth and maturation of follicles and oocytes (50). A recent study demonstrated that mechanical effects caused by internal or external forces can affect follicular activation *via* the Hippo and Akt pathways, involving signaling pathways such YAP, TAZ, PTEN, mTOR, and Foxo3.

### Notch pathway

The Notch signaling pathway is a widely distributed and evolutionarily conserved pathway that controls numerous cellular activities, including cell proliferation, migration, differentiation, and death in both healthy and unhealthy conditions (51). Certain physiological processes in the ovary associated with the Notch pathway are crucial for female reproduction and directly affect female fertility. This pathway consists mainly of ligands and receptors. The receptors are unidirectional transmembrane proteins with numerous intricate structural domains, and extracellular domains of receptors are important for delivering signaling transmitters to cells nearby (52). There are four main types of Notch receptors that control the expression of downstream target genes by interacting with nuclear transcription factors. These receptors are encoded by different genes and have various structural features (53). Their ligands mainly include functional Notch ligands, the Delta-like ligand family of unidirectional integral membrane proteins, and these ligands are specifically distributed and expressed in adult organs (54).

In addition to many mitotic cycles, the Drosophila oocyte maturation process also includes an endoreplication step in which genomic DNA is duplicated without cell division (55). The transition from mitosis to endoreplication is regulated by the Notch pathway (56). In mice, the Notch pathway is expressed temporally and spatially specifically in both germ cells and somatic cells, and inhibition of Notch signaling prevents mouse oocytes from entering meiosis (57). Granulosa cells surround the oocyte and create primordial follicles in the early postnatal period; this is an important step in the development of mammalian follicles. Granulosa cells express the Notch2 receptor gene, which is necessary for the development of follicles (58). Notch2 suppression in granulosa cells reduces oocyte apoptosis, which in turn decreases fertility in mice. Notch2 mutant animals also experienced a drop in the number of primordial follicles. During the maturation of primordial follicles into mature follicles, the volume of oocytes increases, granulocytes proliferate and differentiate, and Notch signaling can promote cell proliferation and regulate ovarian hormone release and ovulation processes (59). When Notch signaling is disrupted, granulosa cell growth is restrained (60).

In a clinical trial, patients with POF were discovered to have mutations in the Notch2 gene, indicating that these mutations may be connected to the emergence of POF (61). In a mouse model of POF, growth hormone was discovered to activate the Notch1 signaling pathway, upregulate Notch1 expression, raise plasma estradiol levels, reduce follicle-stimulating hormone concentrations, alleviate symptoms, and promote ovarian maturation (62). These findings may provide a new target for the treatment of POF.

### KL–KIT pathway

The tyrosine kinase receptor KIT and its ligand cytokine KL mediate signaling between granulosa cells and oocytes and are closely associated with female fertility (63). Follicular development is significantly influenced by the interchange of materials and bidirectional signaling between somatic cells and oocytes. Oocytes play a critical function in the differentiation and proliferation of granulosa cells, and granular cells can provide growth factors to oocytes. KIT, encoded by the c-kit gene, is a tyrosine kinase type III receptor expressed on the surface of hematopoietic stem cells and others. KIT consists of extracellular, transmembrane, proximal, and intracellular structural domains. Five immunoglobulin-like domains make up the extracellular structural domain, while a variable length sequence divides the domain into distal and proximal ends (64). KIT is expressed on the oocyte surface during the whole follicular development process in humans, mice, and rats (65). KL is the ligand of Kit, also known as stem cell factor, and is a growth factor that exists in the form of a monomer. KL is expressed in fibroblasts and endothelial cells and promotes proliferation, migration, differentiation, and survival of germ cells (66). In general, granulosa cells and ovarian epithelial cells generate KL, and follicular membrane cells and oocytes express KIT. KL binds to KIT and forms a dimer, which activates KIT's intrinsic tyrosine kinase activity, causes it to be phosphorylated, and then activates signal transduction molecules in cells. These effects have huge impacts on the production of primordial germ cells in the ovary, the activation of primordial follicles, the survival and growth of oocytes, the proliferation of granulosa cells, and the maintenance of meiosis (67).

The KL–KIT interaction promotes directed migration of primordial germ cells. KL has been shown to support germ cell proliferation *in vitro* culture assays, and the activation of KIT has been found to inhibit germ cell apoptosis by Sakata et al. (68) However, the precise mechanism of action of this pathway is still unknown (68).

This signaling pathway can also play a role in basal follicle activation and early follicular development. After injecting mice with a function-blocking antibody to KIT, Yoshida et al. found that recruitment and growth of primary follicles and proliferation of granules were disturbed (69). Parrott et al. (70) treated the ovaries of 4-day-old rats with culture medium supplemented with KIT blocking antibody, and established a control group at the same time. After 2 weeks of *in vitro* culture, they noticed a decrease in the number of latent primordial follicles in the experimental group and some spontaneous activation of primordial follicles, which supported the involvement of endogenous KL. The proportion of primordial follicles was reduced, whereas the proportion of developing follicles increased when exogenous KL was added to the culture media. Parrott thought KL could activate primordial follicles in mice, but this process is likely to require additional factors (70). A similar study was done by Carlsson et al. using human follicle cells, but they did not find a stimulating effect of KL on the transformation of primordial follicles into primary follicles. They hypothesized that this discrepancy may be caused by species-related differences in rodents and humans. Carlsson et al. (71) posited that KIT and KL are expressed early in follicular development in the human ovary and can control follicle survival during this period, and blocking KIT can induce follicular atresia. However, unlike rodents, exogenous KL cannot improve human follicle survival (71). Jin et al. (72) suggested that KL could promote early follicle development through the mediation of protein kinase C. It could also inhibit oocyte apoptosis by upregulating the anti-apoptotic protein Bcl-2 and downregulating the pro-apoptotic factor Bax (72).

### TGF-β/Smad pathway

Transforming growth factor beta (TGF-β) is a multifunctional cytokine that belongs to the transforming growth factor superfamily, which includes three different isoforms of transforming growth factors (TGF-β 1–3) and many other signaling molecules (73). TGF-β is a doublet composed of two structurally related subunits connected by disulfide bonds. It can be activated by altering the ionic strength or by hydrolysis and excision actions of proteases, forming a serine/threonine kinase complexes with other factors, and attaching to the TGF-β receptor (74). Type 1 receptor (TGF R1) and type 2 receptor (TGF R2) make up the TGF β receptor, and TGF R1 can be blocked by SD208. After binding to TGF-β, the TGF R2 phosphorylates and activates the TGF R1, resulting in a series of signaling responses (75). These responses activate downstream substrates and regulatory molecules, which in turn stimulate the expression of target genes and regulate the activation, proliferation, and differentiation of many cell types as well as the self-renewal of stem cells, thus playing crucial roles in embryonic development and tissue homeostasis (76).

The Smads protein is a key downstream medium of TGF-β signaling, which is a group of structurally similar proteins that are the main signal transduction factors of the TGF-β pathway and are essential for regulating cell growth and development. There are three different isoforms of Smads: combinatorial Co-Smads, inhibitory I-Smads, and receptor-regulated R-Smads (77). The intracellular kinase structure of TGF-R1 phosphorylates R-Smads, exposing nuclear input sequences and building a complex with Co-Smads that can relocate to the nucleus and bind to target genes (78). I-Smads TGF-β signals through a variety of mechanisms, including blocking the binding of R-Smads to TGF R1 and Co-Smads, reducing TGF R1 expression, and obstructing transcription in the nucleus (79). TGF-β slows cell cycle progression in adult cells and stops cells from entering the G1/S phase transition, leading to the induction of apoptosis. The Smad signaling pathway plays a role in the regulation of this phenomenon; this situation is found in epithelial cells of numerous organs (80). Exhaustion of Co-Smads results in endocrine disturbance and increased follicular atresia in the ovary, and overexpression of I-Smads greatly increases the rate of follicular apoptosis (81, 82). Zhu et al. (83) found that thymopentin has a significant therapeutic effect on POF by stimulating Smad signaling, reducing cellular stress damage and inflammatory factors (83).

TGF-β is crucial for the growth of mouse follicles and gonadal tissues. Zheng et al. conducted *in vitro* mouse ovary culture experiments to further understand the role of TGF-β, in which the control group received no treatment, while experimental groups 1 and 2 received TGF R1 and SD208, respectively. The number and shape of follicles were assessed by sectioning the ovaries after 7 days of culture. It was found that the growth of primordial follicles and oocytes in ovaries treated with TGF R1 was inhibited, the number was decreased, and the proliferation of granulosa cells was significantly weakened. By contrast, in the ovaries treated with SD208, oocytes grew faster, had larger cell diameters, a higher percentage of activated cells, and more granulosa. After TGF R1 expression was significantly reduced by si-RNA, as seen by PCR and western blot, it was observed that the proportion of developing follicles in the ovaries increased, the growth was accelerated, and the volume became larger. Therefore, the researchers came to the conclusion that the TGF-β signaling pathway is crucial for maintaining the dormancy of the primordial follicular pool and that the mechanism of action of SD208 may involve activating the ribosomal protein s6 (RPS6) signaling pathway in mouse ovaries, thereby producing an inhibitory effect to the TGF-β signaling pathway (84).

Further, research has shown that progesterone and luteinizing hormone can increase the survival of preovulatory follicles. In a rat model, luteinizing hormone prevented follicular apoptosis by increasing the level of insulin-like growth factor 1 (85). Progesterone acts on nuclear and membrane receptors to prevent granulosa cell death (86). FSH can promote the secretion of progesterone and increase the expression of the luteinizing hormone receptor, and the TGF-β pathway can enhance this process, thereby indirectly improving the survival rate of follicles (87).

### JAK/STAT pathway

The JANUS kinase-signal transducer and the activator of transcription (JAK/STAT) pathway is considered to be one of the most important signaling pathways in cells. It allows extracellular chemical signals to pass through the cell membrane and be delivered to the DNA promoter in the nucleus to control the expression of the corresponding genes. Several growth factors and cytokines, including interferon, interleukin, and colony-stimulating hormone, are involved in this route, which is related to a number of bodily processes, including immunological adaption, tissue repair, inflammatory response, and apoptosis (88).

The pathway is highly conserved in evolution and consists of JAK, STAT, and the receptor–ligand complex. JAK is a non-acceptor tyrosine kinase that is a subgroup of tyrosine kinases that catalyzes the transfer of phosphate groups from nucleoside triphosphate donors to tyrosine subunits in proteins, regulating cell growth, differentiation, proliferation, death, and migration (89). JAK has two similar phosphate structure transfer domains, one of which has a kinase function, while the other has inhibitory effects on the kinase activity of the first domain. A family of intracellular transcription factors called STAT proteins regulates the proliferation, differentiation, and death of cells (90).

When a ligand binds to the receptor in this pathway, the receptor undergoes a conformational change, so that the two domains in JAK are close to each other and phosphorylation occurs, resulting in conformational change that further activates STAT. The activated STAT signal transfers into the nucleus and regulates the transcription of specific genes (91). Zhang et al. (92) investigated the transcriptomic profile of human granulosa cells and found that the JAK/STAT signaling pathway was more strongly expressed and the expression of JAK1 was upregulated in granulosa cells at the primordial follicle stage, suggesting that this pathway may mediate the transition from primordial to primary follicles (92). Ernst et al. (93) performed similar experiments, sequencing RNA samples from human primordial and primary follicles and discovered that the expression of JAK/STAT pathway was significantly downregulated during the transformation from human primordial to primary follicles, indicating that the pathway's expression starts to weaken after completing the role of mediating follicle maturation (93).

### MAPK pathway

The mitogen-activated protein kinase (MAPK) signaling pathway can transmit signals from the extracellular matrix to the nucleus, which is important for cellular communication. MAPK is a group of serine/threonine protein kinases that can be stimulated by extracellular signals such as cytokines and neurotransmitters to regulate cell growth, proliferation, apoptosis, and other activities (94). The core of this pathway is tertiary, which consists of the MAPK kinase activator, MAPK kinase, and MAPK. As a key mechanism for eukaryotic signal transmission, it governs physiological and pathological processes, as well as gene expression (95). After the cell surface receptor binds to mitogen, it stimulates the Ras protein to convert GDP into GTP, then activates the MAPK pathway and intracellular transcription factors. During this process, MAPK is phosphorylated (96).

The MAPK pathway is involved in regulating the processes of meiosis, cytoplasmic maturation, nuclear membrane formation, chromatin condensation, and spindle assembly. It can be triggered to promote the resumption and progress of meiosis. Salamone et al. compared prepubertal calf and adult bovine oocytes and found that calf oocytes lacked developmental capacity. They found that calf oocytes contained higher concentrations of MAPK after comparing the levels of the two. This demonstrates that MAPK can regulate oocyte activation by influencing cytoplasmic maturation (97).

Goudet et al. (98) discovered that after equine oocytes were cultured*in vitro*, the majority of cells were unable to complete meiosis, and MAPK remained in a non-phosphorylated form; however, in mature equine oocytes, MAPK was phosphorylated and possibly had kinase activity (98). Even though the biochemical mechanism is unclear, it seems that the inability of oocytes to complete meiosis may be caused by unphosphorylated MAPK.

To further clarify the role of the MAPK pathway, Zhang et al. (99) cultured porcine oocytes with U0126, an inhibitor of the MAPK pathway, and observed the process of meiosis and the expression of related genes. They discovered that U0126 could prevent the resumption of meiosis. Some mRNAs in the cells displayed high levels of expression; however, these mRNAs experienced a decline in oocytes that completed meiosis normally. This implies that adequate MAPK expression contributes to normal oocyte development, while normal expression of the MAPK pathway promotes the above mechanisms (99). Furthermore, after transferring U0126-treated oocytes to a drug-free medium, the maturation ability of the oocytes was restored, indicating that the blocking effect of U0126 is reversible.

### Multi-pathway collaboration

Activators of the PI3K signaling pathway can stimulate primordial follicles in ovarian tissue to treat POF. PTEN inhibitors and PI3K activators can regulate the PI3K/Akt signaling pathway in follicles, and can activate quiescent follicles in patients with POF to develop into preovulatory follicles (100). Ovarian rupture can influence the Hippo signaling system and stimulate the growth of secondary follicles. Patients with POF may benefit from a combination of these two methods. Using activators of Akt or the PI3K pathway can simulate resting follicles in ovaries, and then inhibit Hippo signaling through ovarian fragmentation, thereby stimulating follicle growth and obtaining mature egg cells (101). Sun et al. (102) found in mice that the use of gonadotropin-releasing hormone (GnRH) agonist can promote the expression of PI3K/Akt signaling pathway, reduce the occurrence of ovarian atresia, and improve ovarian reserve function (102). Li et al. (103) found that Notch, insulin, and other pathways also interact with Foxo3 to make it act as a guardian of the ovarian follicle pool in mammals and a potential determinant of the onset of menopause (103).

By activating the ribosomal protein s6 (RPS6) signaling pathway, which is a downstream signal of mTORC1, the TGF- pathway inhibitor SD208 can induce follicular growth and break the dormant state of follicles (84). Rapamycin has an inhibitory effect on the mTOR pathway. After the stimulatory effect of SD208 on oocyte growth was observed, rapamycin was found to partially inhibit this stimulatory effect and reduce the number of growing follicles. In addition, the TGF-β signaling pathway can activate the PI3K/Akt pathway in many cells, induce phosphorylation of Akt and Foxo3 (104), and activate the mTORC1 signal *via* the PI3K/Akt/TSC2 pathway (105), but these effects may not involve regulating the growth of primordial follicles. *In vitro* activation (IVA) techniques have been investigated in recent years to mobilize residual follicles in POF patients. Initially, IVA disrupted Hippo signaling by disrupting the ovaries, and then the fragments were cultured *in vitro* with an Akt stimulant for 2 days before being transplanted back into the patient. This method has now been developed into a one-step procedure that does not rely on drugs to obtain fertile, mature follicles. When antral follicles develop to the preovulatory stage, mature oocytes are extracted, and embryos are transferred after *in vitro* fertilization with sperm. IVA has been performed in patients with POF, and the follicles can grow and fertilize successfully (106). To date, about 20 live births have been reported using this technique, which is regarded as the most hopeful method to reproduce genetic offspring in patients with POF. Thus, efforts should be made to clarify the mechanism of action and guarantee the safety of this treatment before it is used extensively in the clinic. In a clinical trial, Suada et al. (107) used autologous growth factors to activate damaged ovaries *in vitro*, then transplanted them into patients, and measured hormone levels in patients. They found that there was a correlation between improved hormone levels and the volume of transplanted tissue (107). Further, interference with the Hippo pathway should be done with caution, since dysregulation of it is linked to the development of cancer.

The process of transformation from primordial follicles into mature follicles is complex. There are a number of intercellular and intracellular signaling pathways that interact in various ways to form a complicated network that can respond to many stimuli, but the precise mechanism remains to be further studied.

Despite these new advances, regulating primordial follicle activation and controlling oocyte development *in vitro* are still different for patients with POF. Moreover, current studies are mainly carried out on animal models; thus, due to the differences between human and animal models, the research findings may have limitations.

## Signaling pathway and stem cells

Some signaling pathways may be related to stem cells. The KL-KIT and TGFβ-Smad and other pathways discussed earlier can regulate the self-renewal of stem cells through different mechanisms and play a role in embryonic development and tissue homeostasis. In recent years, stem cell transplantation has become a hotspot in the treatment of POF. The mechanism by which stem cells improve ovarian function is related to the cytokines or exosomes secreted by them, which regulate immunity and promote follicle development while improving the microenvironment of ovarian tissue, and are related to various signaling pathways.

Stem cells are a subtype of cells that maintain an undifferentiated form in both embryonic and adult tissues and can undergo self-renewal and differentiation (108). By participating in the repair of organ damage, stem cells in differentiated organs promote the restoration of organ function. Stem cells can be categorized as embryonic stem cells (ESC), mesenchymal stem cells (MSC), induced pluripotent stem cells (iPSC), spermatogonia stem cells (SSC), ovarian stem cells (OSC), etc. Stem cell therapy can promote ovulation in patients with POF. There are many studies focusing on different protocols for stem cell isolation, purification, and culture for the treatment of POF (108). Different types of stem cells have been used in recent studies to treat POF (Table 1). Studies have shown that human ESCs play an important role in the repair and functional recovery of endometrial damage, and ESCs have become an important tool for cell therapy (109). MSCs can colonize injured ovaries and secrete and release a range of cytokines to restore ovarian function. According to several studies, MSCs may be used to treat infertility brought on by ovarian and endometrial dysfunction (110). Researchers applied bone marrow mesenchymal stem cells (BMSC) therapy in a rabbit model of ovarian dysfunction induced by cyclophosphamide, and they observed that it restored the function and structure of follicles by decreasing gonadotropin levels and increasing estrogen and vascular endothelial growth factor levels (112). Mohamed et al. (113) transplanted BMSC into mice with POF and observed an increase in ovarian volume and weight in mice, which accommodated the development of follicles (113). Liu et al. (114) found that in mice with POF, human menstrual blood mesenchymal stem cells (hMensSCs) can promote the increase in follicle number and restoration of ovarian function, as well as the secretion of estrogen and anti-Mullerian hormone (AMH) (114). A study revealed that the ovarian reserve function of rats' was improved and the number of follicles rose after receiving transplantation human umbilical cord mesenchymal stem cells (hUCMSCs) (115). The hepatocyte growth factor, which is secreted from hUCMSCs, can increase the activity of the PI3K/Akt signaling in dormant oocytes (116). By transplanting hUCMSCs into mice with POF, Lv et al. (120) found that multiple hUCMSCs transplantations had a better effect on the recovery of ovarian function than single hUCMSCs transplantation (120). iPSCs can be differentiated into hormone-sensitive ovarian epithelioid cells, and when these cells are transplanted into a mouse with POF, an increase in estrogen levels and ovarian weight and a decrease in atretic follicles were observed in mice (117). White et al. (121) isolated OSCs from the human ovarian cortex and transplanted them into immunocompromised mice. They found that the cells could induce the generation of mouse follicles and be used to treat POF (121). Vo et al. (122) suggested that this approach, which uses stem cells to generate new oocytes, is particularly useful in patients with POF who have no residual follicles after chemotherapy or radiotherapy (122). Cacciottola et al. (119) demonstrated that adipose tissue-derived stem cells (ADMSCs) exert positive effects on the ovarian reserve, not only by protecting primordial follicles from direct death but also by maintaining their quiescence through modulation of the PI3K/Akt pathway (119). Furthermore, MSCs can release a variety of bioactive molecules that can regulate inflammation and other immune responses, promote endometrial regeneration, and help restore fertility (123, 124). Qu et al. (111) used exosomes secreted by MSCs to treat POF in rats and found that exosomes could regulate the PI3K/Akt/mTOR pathway, promote ovarian angiogenesis, and induce apoptosis of cells (111). Zhao et al. mentioned in a review that exosomes might represent a new treatment method for enhancing decreased fertility in women with POF (125).

**Table 1:** The applications of stem cells in the treatment of POF.

**Type of stem cell**	**Function**	**Authors name**	**Year**	**References**
ESCs	Repair endometrial damage	Wang et al.	2019	(109)
MSCs	Produce cytokines, regulate inflammation	Trounson et al.	2017	(110)
	Secret exosomes to regulate the PI3K/Akt/mTOR pathway, promote ovarian angiogenesis, induce apoptosis of cells	Qu et al.	2022	(111)
BMSCs	Restore function and structure of follicles and increase estrogen and vascular endothelial growth factor levels	Somia et al.	2013	(112)
	Restore ovarian hormone production and reactivate folliculogenesis	Mohamed et al.	2018	(113)
hMensSCs	Increase ovarian weight, normal follicles number, AMH and estrogen level	Liu et al.	2014	(114)
hUCMSCs	Increase ovarian reserve function	Li et al.	2017	(115)
	Increase the activity of the PI3K/Akt signaling in dormant oocytes	Mi et al.	2022	(116)
iPSCs	Differentiate into ovarian epithelioid cells, increase estrogen levels and ovarian weight, decrease atretic follicles	Liu et al.	2013	(117)
OSCs	Induce the generation of follicle	White et al.	2012	(118)
ADMSCs	Protect primordial follicles from direct death, maintain quiescence through modulation of the PI3K/Akt pathway	Cacciottola et al.	2021	(119)

Although the effect of stem cell therapy has been shown in some animals, its clinical application is still severely limited because of the intrusiveness of stem cell collection and possible ethical and immune rejection issues. Further research is therefore needed to explore stem cells that are simple to collect, non-invasive and safe during collection, low in immunogenicity, and do not involve ethical concerns.

## Summary

POF has significant physiological and psychological impacts on women; thus, it is critical to reveal more knowledge about its pathogenic mechanisms and available treatments. It is necessary to have a better understanding of the mechanisms related to follicular pool depletion and to master methods of intervening in ovarian aging. The activation of primordial follicles is a complex but coordinated process that is regulated by multiple factors and pathways. In this review, we focused on describing the roles of PI3K/Akt/Foxo3 signaling pathway, mTOR signaling pathway, etc. in primordial follicle activation, and also discussed other mechanisms that regulate follicle dormancy and activation. Based on our current understanding of follicle activation, the development of drugs that regulate the activation of primordial follicles and target some signaling pathways may provide new possibilities for the treatment of POF. Further studies can also be conducted on other potential regulators that could help provide patients with a more personalized treatment plan. In recent years, many different types of stem cells have been found to have therapeutic effects on POF. For the protection of female fertility, stem cells can be considered treatment options to restore female fertility. In addition, psychological problems caused by impaired fertility also play a certain role in the treatment of POF patients, and the psychological state of patients should also be paid attention to in the treatment of POF (126). Studies have suggested that infertility problems caused by POF often plague the whole family, and while paying attention and treatment to female members of the family, we should also pay attention to the physical and psychological problems of male members (127).

However, there is still much unknown information about the above approaches, and some measures are still limited to the experimental level due to ethical or technical issues. Thus, further research is required. With the advancement of science, technology, and experimental-level strategies, as well as interventions for improving the quality of life of patients with POF through multidisciplinary therapy, we hope to obtain evidence of the safety and efficacy of these methods through large-scale clinical trials.

## Author contributions

XB conceived the study and wrote the manuscript. SW conceived the study, organized, and edited the text. All authors contributed to the article and approved the submitted version.

## Funding

This work was partly funded by National High Level Hospital Clinical Research Funding (BJ-2021-236).

## Conflict of interest

The authors declare that the research was conducted in the absence of any commercial or financial relationships that could be construed as a potential conflict of interest.

## Publisher's note

All claims expressed in this article are solely those of the authors and do not necessarily represent those of their affiliated organizations, or those of the publisher, the editors and the reviewers. Any product that may be evaluated in this article, or claim that may be made by its manufacturer, is not guaranteed or endorsed by the publisher.
